# Genome-Wide Expression Profiles of Hemp (*Cannabis sativa* L.) in Response to Drought Stress

**DOI:** 10.1155/2018/3057272

**Published:** 2018-05-15

**Authors:** Chunsheng Gao, Chaohua Cheng, Lining Zhao, Yongting Yu, Qing Tang, Pengfei Xin, Touming Liu, Zhun Yan, Yuan Guo, Gonggu Zang

**Affiliations:** Institute of Bast Fiber Crops, Chinese Academy of Agricultural Sciences/Key Laboratory of the Biology and Process of Bast Fiber Crops, Ministry of Agriculture, Changsha 410205, China

## Abstract

Drought is the main environmental factor impairing hemp growth and yield. In order to decipher the molecular responses of hemp to drought stress, transcriptome changes of drought-stressed hemp (DS1 and DS2), compared to well-watered control hemp (CK1 and CK2), were studied with RNA-Seq technology. RNA-Seq generated 9.83, 11.30, 11.66, and 11.31 M clean reads in the CK1, CK2, DS1, and DS2 libraries, respectively. A total of 1292 differentially expressed genes (DEGs), including 409 (31.66%) upregulated and 883 (68.34%) downregulated genes, were identified. The expression patterns of 12 selected genes were validated by qRT-PCR, and the results were accordant with Illumina analysis. Gene Ontology (GO) and KEGG analysis illuminated particular important biological processes and pathways, which enriched many candidate genes such as NAC, B3, peroxidase, expansin, and inositol oxygenase that may play important roles in hemp tolerance to drought. Eleven KEGG pathways were significantly influenced, the most influenced being the plant hormone signal transduction pathway with 15 differentially expressed genes. A similar expression pattern of genes involved in the abscisic acid (ABA) pathway under drought, and ABA induction, suggested that ABA is important in the drought stress response of hemp. These findings provide useful insights into the drought stress regulatory mechanism in hemp.

## 1. Introduction

Abiotic stresses, such as drought, high salt, and extremes of temperature, are major environmental factors that can limit plant growth and development. Among various abiotic stressors, drought has the greatest impact on crop culture and world agriculture [1]. Currently, global warming is increasing the frequency and severity of extreme weather events, including drought, worldwide. It is therefore important to improve plant drought tolerance and further understand the relationship between drought stress and water use for plant growth.

Drought stress or water deficit induces a series of morphological, physiological, biochemical, and molecular changes that influence plant growth, development, and productivity. During their long-term evolution, plants have developed three main mechanisms to adapt to drought stress, including drought escape, drought avoidance, and drought tolerance. It is important to understand the genetic basis of these mechanisms of plants encountering a water deficit [2]. Many plant genes responding to drought stress have been identified by molecular and genomic analyses of *Arabidopsis*, rice, and other plants. These genes are classified into two groups according to their putative functional modes. One group contains proteins that are likely involved in abiotic stress tolerance, such as chaperones, late embryogenesis abundant (LEA) proteins, osmotin, mRNA-binding proteins, key enzymes for osmolyte biosynthesis, water channel proteins, sugar and proline transporters, detoxification enzymes, and various proteases. Another group contains regulatory proteins involved in signal transduction and stress-responsive gene expression, including various transcription factors (TFs), protein kinases, protein phosphatases, enzymes involved in phospholipid metabolism, and other signaling molecules, such as calmodulin-binding proteins [3]. Moreover, some of these proteins (LEA, osmotin, zinc finger protein, NAC, WRKY, bZIP, AP2/ERF, MYB, etc.) have been overexpressed in transgenetic plants, and conferred transgenetic lines that enhanced drought tolerance [4]. All these data verify the contribution of these genes to drought stress response.

Plant cell signaling and molecular regulation networks during drought stress have also been investigated and highlighted. Two important pathways of transcriptional networks were found in *Arabidopsis* and rice, the two model plants, when they were grown under drought conditions: an abscisic acid- (ABA-) dependent signaling pathway and an ABA-independent regulatory network mediated by dehydration-responsive element-binding- (DREB-) type TFs [4, 5]. In the first type, the ABA-responsive element (ABRE) is the major *cis*-element and TFs are the master regulators of drought-responsive gene expression, as they control gene expression in an ABA-dependent manner. Additionally, ABA receptors (PYLs), group A 2C-type protein phosphatases (PP2Cs), and SNF1-related protein kinases 2 (SnPK2) were core components and controlled the ABA signaling pathway. In the ABA-independent regulatory network, NAC TFs were also involved in drought stress response, along with DREB TFs [6].

Hemp (*Cannabis sativa* L.) was one of the earliest domesticated crops and is used today in multiple industrial applications, including the production of fiber, foods, and oils [7, 8]. Hemp has been cultivated to produce textile for more than 6000 years in China, and China is currently the largest producer of hemp seed and textiles for domestic use and exports [9]. Drought stress is the main environmental factor that influences hemp production, limiting growth and reducing fiber quality and yield [10–12]. For instance, more than 10 days of consecutive drought increases the incidence of hemp tip dieback over a 1-month period after sowing [13]. Moreover, as the largest developing country with the largest population worldwide, China has begun to face serious water scarcity issues [14]. Therefore, investigating the mechanisms that regulate drought tolerance in hemp is important for drought-tolerant cultivar development in breeding programs. The complete genome and transcriptome sequences of hemp have been reported [15], along with a diversity analysis based on the large-scale development of expressed sequence tag- (EST-) derived simple sequence repeat (SSR) markers [16]. However, the responsive genes and stress regulatory mechanisms of hemp subjected to drought stress remain elusive.

High-throughput sequencing has become a powerful tool in many research fields due to its cost-efficiency and rapidness [17–23]. The data yielded facilitates the development of genetic analyses and functional genomics studies among species, especially for many nonmodel plants. In particular, this technology has been widely used to understand drought stress response in various plants, such as rice [24], wheat [25], maize [26], soybean [27], cotton [28, 29], potato [30], and ramie [18]. In the present study, the transcriptome changes of hemp to drought stress were investigated and potential responsive genes were identified using the Illumina HiSeq™ 2000 platform. As a result, this study presents the first genome-wide expression profile of hemp responding to drought stress. The findings of this study are expected to provide a foundation for a comprehensive understanding of the mechanisms of hemp subjected to drought stress, along with identifying potential drought resistance genes, which can be used to improve the drought tolerance ability of hemp in breeding programs.

## 2. Materials and Methods

### 2.1. Plant Material, Stress Treatment, and RNA Extraction

Hemp cultivar Yunma 1, which is sensitive to drought [31], was used in this study. The hemp seeds were sowed in a pot (31 cm deep × 34 cm diameter) filled with 16 kg soil, 15 seeds per pot. The pots were kept at 26°C ± 1°C, 75 ± 1 RH, and a photoperiod of 14 : 10 (L : D) in the greenhouse of the Institute of Bast Fiber Crops, Chinese Academy of Agricultural Sciences. After seeds germinated, seedlings were thinned out to keep 10 seedlings per pot. Six potted hemp plants were used as drought stress plants (DS), whereas the other six potted plants were used as control plants (CK). Two replicates of CK and DS were designated, which were named CK1 and CK2 and DS1 and DS2, respectively. Watering was withheld at 30 days after sowing in the soil. CK plants were grown under well-watered conditions, where DS plants were treated with drought stress by controlling the relative water content of soil at no more than 20% (severe drought stress) by natural drying [31]. After 7 days, the CK and DS plants were uprooted, and the leaves, roots, stem bast, and stem shoots were collected separately. For abscisic acid (ABA) treatment, plants were collected at 3 and 6 hours after being sprayed 3 times with 100 *μ*M ABA [32]. The sampled tissues were immediately frozen in liquid nitrogen and stored at −80°C until use. Total RNA was extracted from the tissue of CK1, CK2, DS1, and DS2 using TRIzol reagent (Invitrogen, USA) and treated with DNase I (Fermentas, USA) according to the manufacturer's protocol. RNA quality, purity, and integrity were determined by a NanoDrop 2000 spectrophotometer and an Agilent 100 Bioanalyzer.

### 2.2. cDNA Library Construction and Sequencing

Equal amounts of total RNA from each DS and CK sample were pooled together. Then, the poly (A)^+^ RNAs were purified from 20 *μ*g total RNA by oligo(dT) Dynabeads. To avoid priming bias when synthesizing cDNA, the purified mRNA was first fragmented into small pieces. First-strand cDNAs were synthesized using random hexamer primers, and second-strand cDNAs were synthesized using dNTPs, RNase H, and DNA polymerase I. Double-stranded cDNAs were purified by a QIAquick PCR Purification Kit and repaired, and an adenine base was added to the 3′ end. Two different adapters were ligated to the 5′ and 3′ ends, respectively. The ligated fragments were separated on gel and purified. After amplification by PCR, the fragments were separated using electrophoresis and purified. Paired-end sequencing was performed by the Illumina sequencing platform (HiSeq™ 2000) at Biomarker Technologies Co., Ltd, Beijing, China (http://www.biomarker.com.cn/) according to the manufacturer's instructions (Illumina, San Diego, CA).

### 2.3. Data Processing and Mapping to Reference Genome

Sequencing errors usually bring difficulties to subsequent analyses. Therefore, first all reads with adaptor contamination were discarded. Then, the low-quality reads with more than 10% ambiguous “N” sequences were ruled out. Finally, the low-quality reads (*Q*value < 20) were removed. *Q*30 is equivalent to the probability of an incorrect base call 1 in 1000 times. Clean reads were submitted to Gene Expression Omnibus in NCBI with the GEO accession number GSE56964. RNA-Seq data was assessed by saturation, duplicate reads, and gene coverage analysis, using RSeQC software (http://rseqc.sourceforge.net/). High-quality reads were mapped to the *C. sativa* (strain Purple Kush) genome (GenBank accession no. AGQN00000000) [15] using TopHat2 (http://ccb.jhu.edu/software/tophat/index.shtml). After aligning reads to the genome, Cufflinks (http://cole-trapnell-lab.github.io/cufflinks/) was used to assemble aligned reads into transcripts.

### 2.4. Identification of Differentially Expressed Genes (DEGs)

Differentially expressed genes were identified by comparing the abundance of the same transcript in CK and DS samples. The hemp genome sequences described above were used as a reference. The number of clean reads mapped to the reference genome was used to calculate gene expression levels via RSEM (http://deweylab.github.io/RSEM/). The FPKM (fragments per kilobase of exon per million mapped reads) value of each transcript was used to represent the gene expression level. Differentially expressed genes (DEGs) were identified using DESeq2 [33]. All statistical tests were corrected for multiple testing with the Benjamini–Hochberg false discovery rate (FDR < 0.01). DEGs were defined as being significantly differentially expressed along with an FDR < 0.01 and a more than two-fold change (>1 or <−1 in log 2 ratio value).

### 2.5. Functional Classification and Annotation for DEGs

Unigenes were annotated using BLASTx alignment (*E*value < 10^−5^) against five databases, including NCBI nonredundant protein database (Nr, http://www.ncbi.nlm.nih.gov), Swiss-Prot (http://www.expasy.ch/sprot), Gene Ontology (GO, http://wego.genomics.org.cn/cgi-bin/wego/index.pl), Clusters of Orthologous Groups (COG, https://www.ncbi.nlm.nih.gov/COG/), and the Kyoto Encyclopedia of Genes and Genomes (KEGG) pathway database [34]. Nr and GO annotation was performed using the Blast2GO program [35]. GO classification was performed using WEGO software to view the distribution of gene functions.

### 2.6. Pathway Enrichment of DEG

Pathway enrichment analysis based on the KEGG pathway database (http://www.genome.jp/kegg) was used to identify markedly enriched metabolic pathways or signal transduction pathways in differentially expressed genes, comparing the whole genomic background. The following formula was used for the calculation of

(1) $$\begin{array}{c} p=1-\overset{m-1}{\underset{i=0}{\sum }}\frac{\left(\begin{array}{c} M\\ i \end{array}\right)\left(\begin{array}{c} N-M\\ n-i \end{array}\right)}{\left(\begin{array}{c} N\\ n \end{array}\right)}, \end{array}$$

where *N* is the number of all genes with a KEGG annotation, *n* is the number of DEGs in *N*, *M* is the number of all genes annotated to specific pathways, and *m* is the number of DEGs in *M*.

### 2.7. Quantitative Real-Time PCR Analyses

For quantitative real-time PCR (qRT-PCR) analysis, total RNA was extracted from the tissue of CK and DS hemp using TRIzol reagent (Invitrogen, USA) and treated with DNase I (Fermentas, USA) according to the manufacturer's protocol. Subsequently, 2 *μ*g total RNA was reverse-transcribed into cDNA. qRT-PCR was performed in an iQ5 multicolor real-time PCR detection system (Bio-Rad, USA) with a 20 *μ*L reaction system containing 10 *μ*L iQ™ SYBR Green Supermix (Bio-Rad, USA), 10 pmol each of the forward and reverse gene-specific primers, and 5 *μ*L diluted cDNA (1 : 50). Gene-specific primers (Table S1) were designed using Primer Premier 5 software. The hemp actin gene was selected as an internal control to normalize the total amount of cDNA present in each reaction. In brief, following a denaturation step at 95°C for 30 s, the amplification was carried out with 40 cycles at a melting temperature of 95°C for 10 s and an annealing temperature of 55°C for 30 s. The qRT-PCR experiments were performed in triplicate.

## 3. Results

### 3.1. Sequencing and Mapping Reads to the Hemp Reference Genome

**Figure 1:**
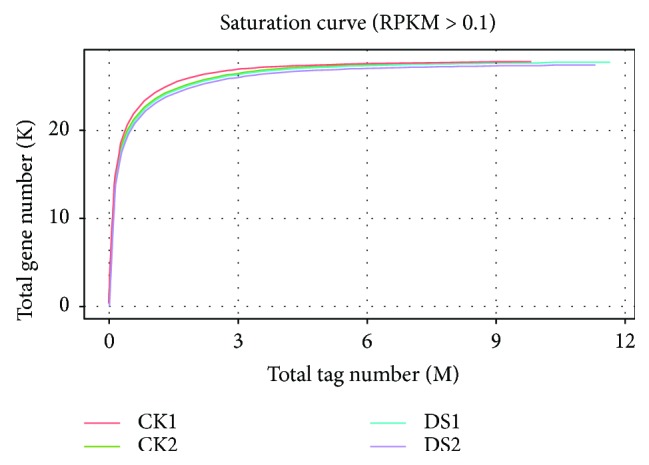
Saturation evaluations of CK and DS hemp.

Four samples (two control samples, CK1 and CK2, and two drought stress samples, DS1 and DS2) were collected separately. After filtering, a total of 44.10 M clean reads were obtained in the CK and DS libraries (Table 1). The number of clean reads for CK1, CK2, DS1, and DS2 was 9.83 M, 11.30 M, 11.66 M, and 11.31 M, respectively. The GC contents of the four libraries ranged from 44.18% to 44.30%. In addition, the *Q*30 for all libraries were all over 90%, indicating the high accuracy and quality of the sequencing data. To annotate the function of the reads, Bowtie was used to align the reads with the published hemp genome. The mapped reads from the four hemp samples were 8.61 M (87.61%), 9.94 M (87.94%), 10.25 M (87.96%), and 9.92 M (87.65%), respectively. These results indicate that reliable sequence alignment results were obtained for gene analysis. Saturation of the library was determined by checking the number of detected genes. When the sequencing level reached 6 M or more, only a few new genes were detected in the four libraries (Figure 1), indicating all four libraries were sequenced to saturation.

### 3.2. Comparison of the Gene Expression Level between CK and DS Libraries

For analysis of gene expression levels, the number of unambiguous clean reads for each gene was calculated and normalized to RPKM. Distribution and density distribution of RPKM showed there to be few different genes among the samples (Figure S1). Additionally, the RPKM values for all genes were compared between both the two DS replicates and the two CK replicates. There were significant correlations between the two CK replicates and the two DS replicates, with Pearson's correlation coefficients of the CK and DS groups of 0.82 and 0.99, respectively. Scatter diagrams were created, in which the logarithmic RPKM values of each gene in the two replicates of each gene, in the two replicates of each treatment, were assigned as coordinate values of two axes, showed that all data points were distributed in the region of the diagonal (Figure S2). These results suggested that the abundances and expression levels of genes in the two DS libraries and in the two CK libraries were similar.

**Figure 2:**
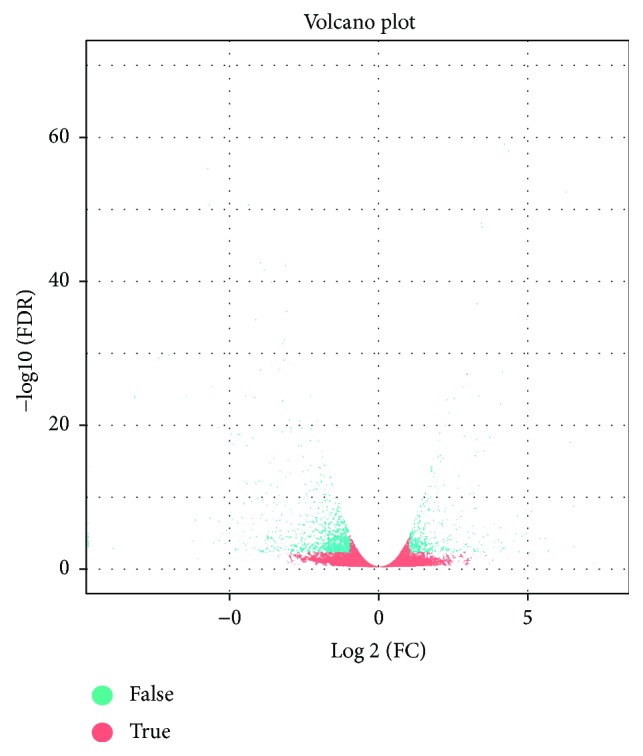
Volcano analysis of differentially expressed genes (DEGs) between CK and DS hemp. In the volcano plot, statistical significance (log_10_ of *P* value; *y*-axis) has been plotted against log_2_-fold change (*x*-axis).

The expression levels of DEGs between the two libraries were evaluated by detecting the sequence frequencies. Following data analysis, only transcripts with FDR < 0.01 and fold change > 2 were selected to represent differentially expressed genes. Based on these criteria, a total of 1292 DEGs were identified, including 409 (31.66%) upregulated genes and 883 (68.34%) downregulated genes (Figure 2, Table S2). Of the total DEGs, 126 showed more than 20-fold (∣log2FC∣ > 4.32) the expression differences, including 43 upregulated genes and 83 downregulated genes.

### 3.3. Functional Annotation and Classification of DEGs

**Figure 3:**
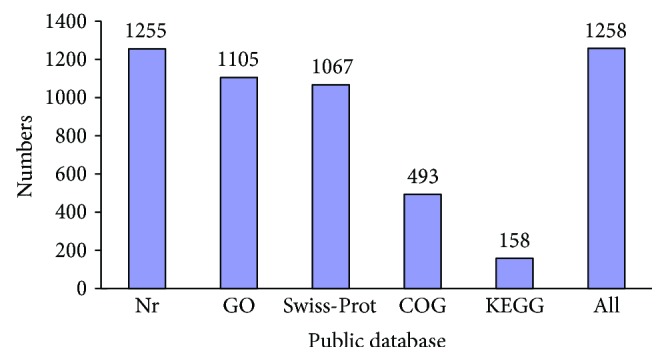
Numbers of differentially expressed genes (DEGs) annotated in five public databases.

To investigate the function of the 1292 DEGs, five databases were used to screen for sequence similarities. These databases included the NCBI Nr database, the Swiss-Prot protein database, the GO database, the KEGG database, and the COG database. The results indicated that 1255 (97.14%), 1067 (82.59%), 1105 (85.53%), 158 (12.23%), and 493 (38.16%) DEGs exhibited significant similarity to known genes in the five databases, respectively (Figure 3). Overall, 1258 (97.37%) DEGs, including 394 (31.32%) upregulated and 864 (68.68%) downregulated DEGs, exhibited similarities to known genes in the five databases (Table S2). This information provided a good reference for gene function analysis.

**Figure 4:**
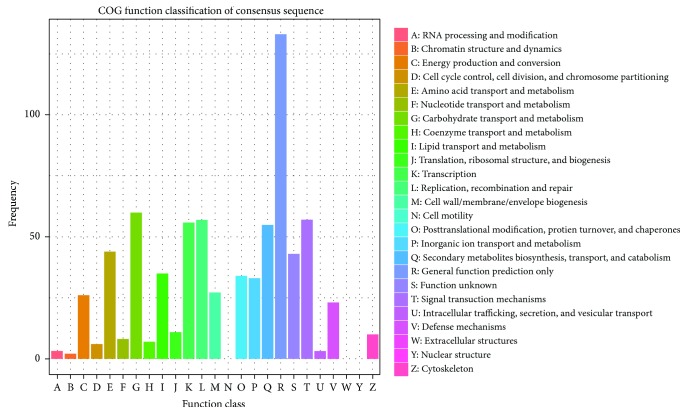
Clusters of orthologous group (COG) classification of differentially expressed genes (DEGs) in drought-stressed hemp.

We used the COG database for genome-scale analysis of protein functions and evolution. A total of 416 DEGs were assigned to the 22 COG classifications, of which 6 COG classifications contained more than 50 DEGs (Figure 4). The 6 largest COG categories were “general function prediction only” (R, 133 DEGs, 31.197%), “carbohydrate transport and metabolism” (G, 60 DEGs, 14.92%), “replication recombination and repair” (L, 57 DEGs, 13.70%), “signal transduction mechanisms” (T, 57 DEGs, 13.70%), “transcription” (K, 56 DEGs, 13.46%), and “secondary metabolite biosynthesis, transport and catabolism” (Q, 55 DEGs, 13.22%).

### 3.4. GO and KEGG Enrichment of DEGs

**Figure 5:**
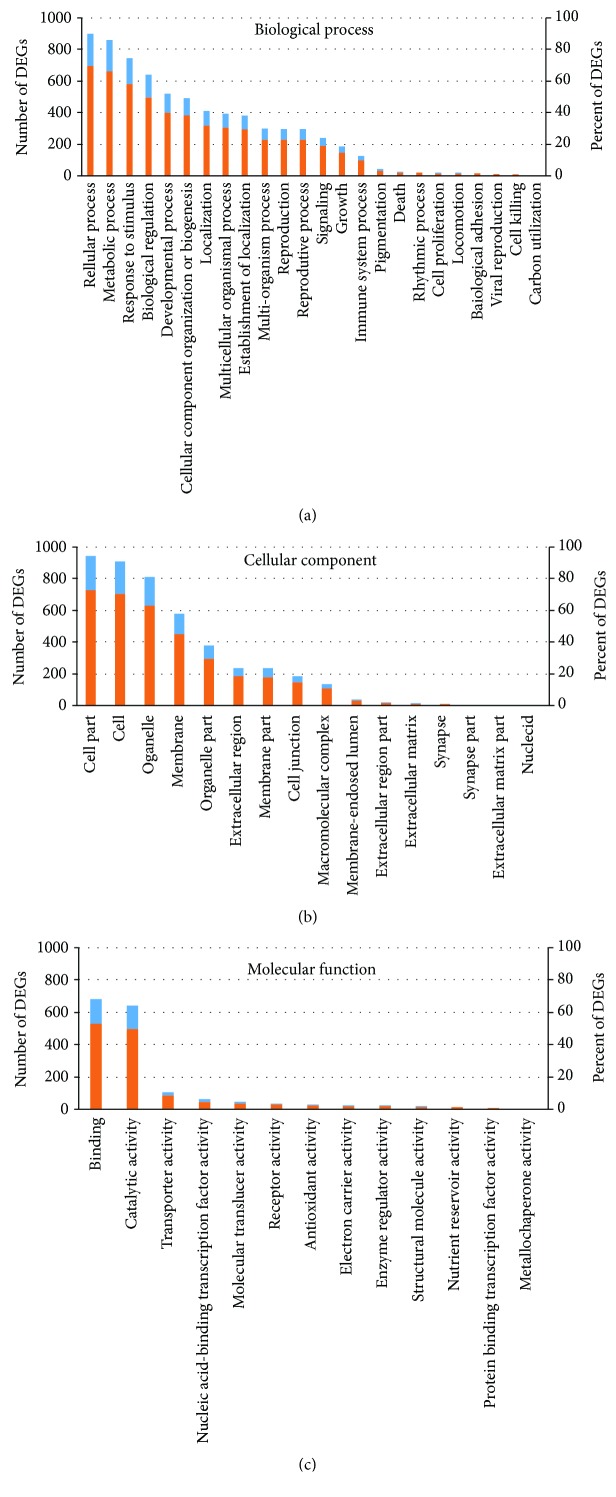
Gene Ontology (GO) enrichment of differentially expressed genes (DEGs) in drought-stressed hemp. The results are summarized in three main categories: biological processes (a), cellular components (b), and molecular functions (c). The blue (corresponding to the left *y*-axis) and orange columns (corresponding to the right *y*-axis) represent the number and percent of DEGs in that category, respectively.

The GO database is a tool for the unification of biology, providing structured, controlled vocabularies and classifications that cover several domains of molecular and cellular biology. Using GO analysis, a total of 1106 DEGs matching known genes were assigned to 53 functional terms of GO for biological processes, cellular components, and molecular function categories (Figure 5). The three GO categories enriched 1011, 997, and 933 DEGs, respectively (Table S3). Among these GO terms, “cellular process” (898 DEGs, 69.50%), “metabolic process” (855 DEGs, 66.18%), “response to stimulus” (744 DEGs, 57.59%), “biological regulation” (639 DEGs, 48.46%), and “developmental process” (514 DEGs, 39.78%) were the dominant biological process terms; “cell part” (937 DEGs, 72.52%), “cell” (908 DEGs, 70.28%), “organelle” (806 DEGs, 62.38%), and “membrane” (577 DEGs, 44.66%) were the dominant cellular component terms; and “binding” (680 DEGs, 52.63%) and “catalytic activity” (638 DEGs, 49.38%) were the most abundant molecular function terms. Additionally, the “transporter activity” and “receptor activity” GO terms enriched 104 (2.55%) and 33 (8.05%) DEGs, respectively.

Biological pathways play a key role in advanced genomics studies. The influence of drought stress on biological pathways in hemp was analyzed by enrichment analysis of DEGs. A total of 77 pathways (210 DEGs) were possibly influenced, out of which 11 pathways were significantly influenced (*P* value < 0.05) (Table 2). Among the 11 pathways, the top five most significantly influenced pathways were plant hormone signal transduction (ko04075), phenylpropanoid biosynthesis (ko00940), cyanoamino acid metabolism (ko00460), carbon fixation in photosynthetic organisms (ko00710), and plant hormone signal transduction (ko00475). The plant hormone signal transduction pathway enriched the most DEGs (15), distributed in the abscisic acid (ABA) (8), auxin (5), jasmonic acid (1), and salicylic acid (1) metabolic pathways (Table S4). Of the 15 DEGs that were differentially regulated by drought stress, 7 DEGs were upregulated and 8 DEGs were downregulated. Interestingly, all the DEGs in the auxin, jasmonic acid, and salicylic acid metabolic pathways were significantly downregulated. In contrast, the DEGs in the ABA metabolic pathway were significantly upregulated. The photosynthesis pathway was the most significantly enriched pathway, with the lowest *P* value. All DEGs enriched by the photosynthesis pathway (ko00195), and photosynthesis–antenna proteins (ko00196), were downregulated, indicating that photosynthesis in drought-stressed hemp was reduced. All DEGs enriched in cyanoamino acid metabolism, and most of the DEGs enriched by phenylpropanoid biosynthesis and carbon fixation in photosynthetic organisms, were downregulated, with ratios of 12/14 and 8/9, respectively. These results suggest the three pathways were significantly suppressed.

### 3.5. Identification of Genes Responding to Drought Stress

To identify drought stress-responding genes in hemp, DEGs with the following characteristics were analyzed: (1) involvement in significantly influenced GO terms and KEGG pathways, (2) connection to a biological process related to water deprivation and desiccation and stomatal movement, and (3) exhibition of a higher fold change (>20) in differential expression. Of the significantly influenced biological processes (*P* value < 0.05) related to water stress, response to water deprivation (GO: 0009414), response to desiccation (GO: 0009269), and regulation of stomatal movement (GO: 0010119) enriched 94, 22, and 29 DEGs, respectively. Moreover, the DEGs with a high ratio or higher fold change in expression included peroxidase (POD), glycine-rich protein (GRP), expansin, nitrate transporter, *β*-amylase 1 (BAM), laccase, protein phosphatase 2C (PP2C), and a variety of transcription factors (TFs) (Table S5). Among the significantly enriched pathways, DEGs with a high ratio or higher fold change in expression included POD, inositol oxygenase, *β*-glucosidase, and (R)-mandelonitrile lyase (Table S4).

A total of 51 transcription factors (TFs), including 20 upregulated and 31 downregulated TFs, were identified to be significantly differentially expressed under drought stress (Table 3). Among these TFs, all 3 *NAC* genes, 4/7 *MYB* genes, and 3/5 *HD-Zip* genes were upregulated, while all 5 *B3* (B3 domain-containing transcription factor), 2 *KNOX* genes, 2 C2H2L genes, and 4/6 bHLH genes were downregulated (Table S6).

### 3.6. Analysis of DEGs by qRT-PCR

**Figure 6:**
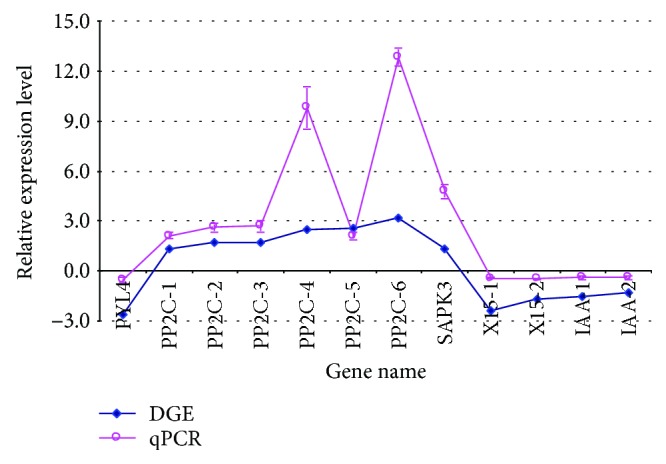
Validation of the expression of drought stress-induced genes by qRT-PCR. Data from qRT-PCR are means of three replicates, and bars represent standard error. PYL4: abscisic acid receptor PYL4 (JP449530); PP2C-1: protein phosphatase 2C (JP474397); PP2C-2: protein phosphatase 2C (JP473042); PP2C-3: protein phosphatase 2C (JP478284); PP2C-4: protein phosphatase 2C (JP477418); PP2C-5: protein phosphatase 2C (JP469562); PP2C-6: protein phosphatase 2C (JP455237); X15-1: auxin-induced protein X15 (JP473847); X15-2: auxin-induced protein X15 (JP472037); IAA-1: indole-3-acetic acid amido synthetase (JP480028); IAA-2: indole-3-acetic acid-induced protein ARG7 (JP461329); SAPK3: serine/threonine-protein kinase (JP477935). Pearson correlation coefficient (*R* = 0.777) analysis showed strong correlation between the qRT-PCR and RNA-Seq data.

**Figure 7:**
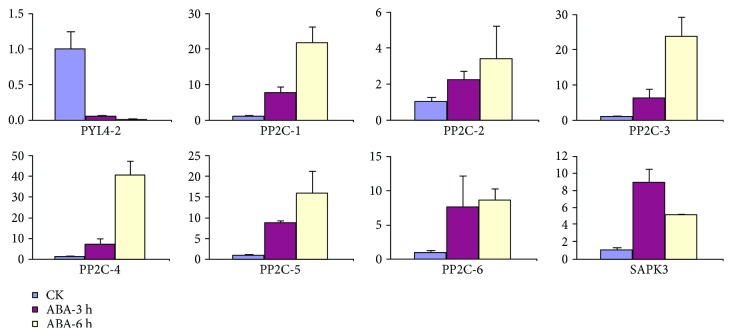
Expression profiles of drought stress-induced genes after ABA treatment determined by qRT-PCR. Data from qRT-PCR are means of three replicates, and bars represent standard error. PYL4: abscisic acid receptor PYL4 (JP449530); PP2C-1: protein phosphatase 2C (JP474397); PP2C-2: protein phosphatase 2C (JP473042); PP2C-3: protein phosphatase 2C (JP478284); PP2C-4: protein phosphatase 2C (JP477418); PP2C-5: protein phosphatase 2C (JP469562); PP2C-6: protein phosphatase 2C (JP455237); SAPK3: serine/threonine-protein kinase (JP477935).

To validate DEGs identified by RNA-Seq, the expression pattern of 12 genes in drought-stressed hemp was studied by qRT-PCR. Among these genes, 8 (*PYL4*, *PP2C-1* to *PP2C-6*, and *SAPK3*) were ABA metabolism-related genes, and 4 (*X15-1*, *X15-2*, *IAA-1*, and *IAA-2*,) were auxin metabolism-related genes. The results showed that 7 genes were upregulated and 5 genes were downregulated, and this trend in the change of gene expression was consistent with that detected by RNA-Seq (Figure 6). In addition, there was a strong correlation (Pearson correlation coefficient of *R* = 0.777) between the qRT-PCR and RNA-Seq data. To investigate the relevance of the 8 ABA metabolism-related genes to drought stress, expression profiles of these genes at 3 h and 6 h after ABA treatment in hemp were also studied by RT-PCR. As shown in Figure 7, the expression of 7 genes of *PP2C* and *SAPK3* were upregulated, and a *PYL4* gene was suppressed in ABA-treated hemp plants (Figure 7), which was in accordance with analysis obtained from drought-stressed hemp. These results signify that ABA may play an important role in regulating the response of hemp to drought stress.

## 4. Discussion

### 4.1. Identification of 1292 Genes Responded to Drought Stress in Hemp

Drought stress is one of the most important environmental factors limiting hemp growth; however, the mechanism of hemp tolerance to drought stress remains unclear. China, as the country with the largest population worldwide, needs more irrigable farmland to grow grain crops to ensure food security; thus, hemp has been mainly planted on un-irrigable dry land and hill slopes. It is vital to study the drought stress response mechanisms of hemp to know how cultivars have adapted to thrive under adverse conditions. However, few studies have focused on identifying the drought response genes and regulatory mechanism of hemp. In this study, a total of 1292 potential drought stress-responsive genes were identified in hemp using RNA-Seq technology. Out of these genes, 1258 (97.37%) were annotated by the five widely used databases (Table S2). These potential drought stress-responsive genes are expected to be useful for investigating the molecular mechanisms of hemp drought tolerance.

### 4.2. Dramatic Changes of Hemp in Response to Drought Stress

Drought stress induces a range of physiological and biochemical responses in plants. These responses include the repression of cell growth and photosynthesis and the activation of respiration [36]. Similar responses occurred in hemp. For example, most DEGs enriched in significantly influenced biological processes involved in cell growth were downregulated (cell wall modification involved in multidimensional cell growth, GO:0042547; multidimensional cell growth, GO:0009825), photosynthesis (photosynthesis, light reaction, GO:0019684; photosynthesis, GO:0015979; photosynthesis, light harvesting, GO:0009765; regulation of photosynthesis, GO:0010109) (Table S2). Additionally, almost all the genes related to photosynthesis and the photosynthesis-related pathways were downregulated. Photosynthesis is a process used by plants and other organisms to convert light energy into chemical energy, which provides plants with the food and energy they need to grow. Our results indicated that drought noticeably altered energy metabolism to avoid damaging hemp. This phenomenon has also been documented in other higher plants [37]. It is shown that some plant growth-related processes were suppressed and a series of responses were activated to facilitate the survival of hemp under drought stress.

Most DEGs involved in biosynthesis of secondary metabolites (phenylpropanoid biosynthesis) and amino acid metabolism (phenylalanine metabolism and cyanoamino acid metabolism) were significantly downregulated, suggesting that these pathways were also suppressed. Among these DEGs, 16 of 22 were peroxidase homologs of hemp. POD is an important antioxidant enzyme in ROS metabolism. ROS is always enhanced when plants suffer drought stress [38]. A decrease of POD activities in drought-stressed hemp implied the decrease in scavengers of ROS, thus resulting in the steady state of cellular ROS breaks and an increase of ROS in hemp.

Interestingly, in the “ascorbate and aldarate metabolism” and “inositol phosphate metabolism” pathways, more DEGs (5 and 4, resp.) were upregulated and limited DEGs (1 and 1, resp.) were downregulated. The upregulated genes contained 3 inositol oxygenase homologs and an inositol-3-phosphate synthase homolog of hemp (Table S5). Among the two pathways, the same upregulated pathway was D-glucuronate synthesis. D-glucuronate is directly synthesized from inositol and is used in the production of cell wall components, glycoproteins, gums, and mucilage. Inositol itself and these methylated derivatives increase in some animal and plant cells, in association with high external NaCl concentrations and dehydration [4, 39]. *OsMIOX*, a myo-inositol oxygenase gene, has been shown to improve drought tolerance of rice [40]. Thus, D-glucuronate synthesis and inositol may be important in the hemp drought response.

### 4.3. Changes of Genes Involved in Sucrose/Starch Synthesis and Cell Wall Plasticity

It has been reported that genes involved in sucrose\starch metabolism are always affected by water stress during grain filling in some crops [41, 42]. In the drought-stressed hemp, the starch and sucrose metabolism (ko00500) pathway (enriched 3 upregulated and 7 downregulated DEGs) was not significantly influenced (*P* value > 0.05). No starch synthesis-related enzyme, such as sucrose transporter, sucrose synthase, starch synthase, and branching enzyme of hemp, were significantly differentially expressed in drought-stressed hemp. However, three *β*-amylase 1 homologs of hemp were upregulated. During osmotic stress, starch can be degraded by stress-activated *β*-amylase 1 to release sugar and sugar-derived osmolytes [43]. Simply put, under drought stress, starch degradation was increased in hemp plants.

Plant cell walls are complex structures composed of cellulose, hemicellulose, pectin, protein, lignin, and various inorganic compounds. Cell wall plasticity has been reported to be related to activities of xylosyltransferase and cellulose synthesis inhibitors [44]. The content of xyloglucan in a cell well affects the mechanical properties of a plant [45]. GO analysis showed that most of the DEGs involved in xyloglucan and cellulose biosynthetic processes, and metabolic processes (GO:0030243, GO:0030244, GO:0010411, GO:0009969, GO:0030244, and GO:0052541), were downregulated. In addition, a zeatin O-xylosyltransferase and a xylosyltransferase 1 homolog of hemp were down- and upregulated, respectively, under drought stress, while the fold change of the former was higher than that of the latter. These data suggest that synthesis and metabolism of xyloglucan and cellulose may be reduced, and the cell wall plasticity was weakened.

### 4.4. Transcription Factors Responding to Drought Stress

Transcription factors are master regulators that control gene clusters. Recent studies demonstrated that many TFs, such as *AP2/ERF*, *NAC*, *HD-Zip*, and *WRKY*, have important roles in response to abiotic stresses in plants [46, 47]. In the drought-stressed hemp, 51 DEGs of families including *bHLH*, *MYB*, *NAC*, *WRKY*, and *AP2/ERF* were identified. Among these TFs, all 5 B3 domain-containing transcription factors were significantly downregulated (>20-fold). Although there was still no substantial evidence that B3 TFs were involved in plant drought tolerance, they may play some role in drought adaptation. Abundant studies have reported that overexpression of genes of the *NAC* family can enhance drought tolerance of plants [48–50]. The observation that all 3 *NAC* TFs were upregulated indicated that these 3 NAC homologs of hemp were likely involved in drought resistance. Further analyses of these transcription factors may provide new insight into the complex regulatory gene networks in response to drought stress in hemp.

### 4.5. ABA May Be a Key Regulation Factor in Hemp Responding to Drought

Plant hormones are important for regulating developmental processes and signaling networks in plant responses to biotic and abiotic stresses, including drought [51, 52]. ABA is critical to osmotic stress regulation among plant hormones; in fact, it is sometimes defined as a stress hormone because of its rapid accumulation and mediation in plant survival when subjected to various stresses. In our study, eight genes in ABA biosynthesis were noticeably influenced by drought stress, including PYL4, PP2C, and SAPK3 (Figure 6). The Illumina sequencing results were also confirmed by qRT-PCR (Figure 6) and were in accordance with those in ABA-treated hemp plants (Figure 7). PYL-PP2C-SnRK2 families function as the core components of ABA signaling, PYLs are ABA receptors; PP2Cs and SnPKs are important negative and positive regulators of ABA signaling, respectively [53, 54]; and SAPK3 belongs to the SnRK2 family [55]. Overexpression of some PYL, PP2C, and SAPK2 genes significantly increases or decreases drought tolerance in transgenic plants [56–58]. Moreover, the ABA receptor PYL5 can inhibit the activity of clade A PP2Cs [59], and PP2CA together with ABI1 inhibits SnRK2.4 activity and regulates plant responses to salinity [60]. These data clarify the function and nature of the cascade relationship of these genes in the stress response of plants. In this study, the expression of PYL4 was downregulated, while PP2Cs and SAPK3 were both upregulated; PP2Cs and SAPK3 displayed the same expression trends in both drought-stressed and ABA-treated hemp plants. Similar findings were also reported in drought- or salinity-stressed tomato and jute plants [23, 61] and ABA-treated tomato plants [62]. Additionally, a gene encoding PYL4 was also downregulated in drought-stressed potato plants [30]; *CsPYL3* was downregulated, although *CsPYL1*, *CsPYL2*, *CsPP2C2*, and *CsSnKR2.2* were upregulated in roots and stems of cucumber seedlings under drought conditions [63]. These data indicated that different numbers of PYLs, PP2Cs, and SnKR2s display different responses to ABA or drought stress. ABA is also important for the regulation of stomatal movement and closure in response to drought [64, 65]. Taken together, ABA may play an important role in regulating stomata closure, reducing water evaporation, and launching the resistance response in hemp.

The plant hormone auxin is essential to many aspects of plant growth and development. In our study, transcripts in auxin biosynthesis pathways clearly decreased in hemp under drought stress (Figure 6). The GH3 gene encodes IAA-amido synthetase, which acts as an auxin-responsive gene, and can help to maintain auxin homeostasis by conjugating excess IAA to amino acids [66]. A rice GH3 gene, OsGH3-2, is involved in negatively regulating ABA levels and drought tolerance [67]. The GH3 homolog of hemp was significantly downregulated under drought stress. These results demonstrate that decreased IAA expression is responsible for repressed cell enlargement, photosynthesis, and plant growth during water stress in hemp.

## 5. Conclusions

This study presents, for the first time, the characterization of genome-wide expression profiling of hemp in response to drought stress. A total of 1292 DEGs were identified, including 409 upregulated genes and 883 downregulated genes. Some genes, including POD, expansin, *NAC*, and *B3* TFs, were shown to be significantly enriched by GO and KEGG analyses, and they may play important roles in hemp adaptation or tolerance to the osmotic and oxidative stresses caused by drought stress. These genes may contribute to further study of the drought stress tolerance of hemp. In addition, we demonstrated that ABA and auxin are crucial in the response of hemp to drought stress. These results are expected to help improve our understanding of the drought stress regulatory mechanism of hemp and improve the drought tolerance ability of the crops.

## Figures and Tables

**Table 1:** Quality of Illumina sequencing and the statistics of reads.

Sample	Total reads (M)	Total base (GB)	GC percent (%)	Q30 percent (%)	Mapped reads (%)	Uniquely mapped reads (%)
CK1	9.83	1.99	44.20	90.03	8.61 (87.61%)	7.08 (82.21%)
CK2	11.30	2.28	44.30	90.06	9.94 (87.94%)	8.18 (82.33%)
DS1	11.66	2.35	44.22	90.07	10.25 (87.96%)	8.41 (82.03%)
DS2	11.31	2.29	44.18	90.01	9.92 (87.65%)	8.08 (81.51%)
Total	44.10	8.91				

**Table 2:** List of pathways significantly enriched in DEGs (*P* < 0.05).

KEGG pathway	Number of genes	Number of DEGs	Up	Down	*P* value	Pathway ID
Photosynthesis	62	14	0	14	1.47*E* − 8	ko00195
Phenylpropanoid biosynthesis	132	14	2	12	1.67*E* − 4	ko00940
Cyanoamino acid metabolism	55	8	0	8	5.45*E* − 4	ko00460
Carbon fixation in photosynthetic organisms	84	9	1	8	2.35*E* − 3	ko00710
Plant hormone signal transduction	199	15	7	8	3.57*E* − 3	ko04075
Photosynthesis—antenna proteins	25	4	0	4	1.01*E* − 2	ko00196
Ascorbate and aldarate metabolism	45	5	4	1	1.90*E* − 2	ko00053
Nitrogen metabolism	66	6	0	6	2.63*E* − 2	ko00910
Phenylalanine metabolism	108	8	1	7	3.34*E* − 2	ko00360
Pentose and glucuronate interconversions	54	5	0	5	3.85*E* − 2	ko00040
Inositol phosphate metabolism	74	6	5	1	4.28*E* − 2	ko00562
Total number	904	94	20	74		

**Table 3:** Summary of DEGs annotated as transcription factor.

Gene family	Number of genes		
	Total	Downregulated	Upregulated
AP2/ERF	2	1	1
B3	5	5	0
bHLH	6	4	2
bZIP	3	2	1
C2H2L	2	2	0
Dof	1	1	0
HD-Zip	5	2	3
KNOX	2	2	0
LHY	1	0	1
MYB	7	3	4
MYB1R1	2	1	1
NAC	3	0	3
NF-YA	2	1	1
SPB	1	0	1
Trihelix	1	0	1
Wox	1	1	0
WRKY	2	1	1
YABBY	1	1	0
ZF (CO-like)	3	2	1
ZF-HD	1	1	0
Total	51	30	21

## Supplementary Materials

Supplementary 1 Figure S1: RPKM distribution and RPKM density distribution of different genes in the DS and CK hemp. A: box-plot of RPKM. *x*-axis means sample names, and *y*-axis means log10(RPKM). B: RPKM distribution. *x*-axis represent log10(RPKM), and *y*-axis represents gene density. E1, E2, E3, and E4 represent DS1, DS2, CK1 and CK2, respectively. Figure S2: correlations between two repeats of the CK and DS samples. E1 and E2 represent DS1 and DS2, and E3 and E4 represent CK1 and CK2, respectively. The logarithmic values of (FPKM) for each gene in the two replicates were assigned as coordinate values of the two axes.

Supplementary 2 Table S1: the sequence of primers used for qRT-PCR analysis.

Supplementary 3 Table S2: summary of DEGs annotated by 5 databases.

Supplementary 4 Table S3: summary of GO-enriched DEGs.

Supplementary 5 Table S4: details of 11 significantly enriched KEGG pathways in DEGs.

Supplementary 6 Table S5: GO terms responding to drought stress.

Supplementary 7 Table S6: details of DEGs annotated as transcription factor.

## References

[1] Vinocur B., Altman A. (2005). Recent advances in engineering plant tolerance to abiotic stress: achievements and limitations.

[2] Zhang Q. (2007). Strategies for developing Green Super Rice.

[3] Yamaguchi-Shinozaki K., Shinozaki K. (2006). Transcriptional regulatory networks in cellular responses and tolerance to dehydration and cold stresses.

[4] Todaka D., Shinozaki K., Yamaguchi-Shinozaki K. (2015). Recent advances in the dissection of drought-stress regulatory networks and strategies for development of drought-tolerant transgenic rice plants.

[5] Huang G. T., Ma S. L., Bai L. P. (2012). Signal transduction during cold, salt, and drought stresses in plants.

[6] Nakashima K., Yamaguchi-Shinozaki K., Shinozaki K. (2014). The transcriptional regulatory network in the drought response and its crosstalk in abiotic stress responses including drought, cold, and heat.

[7] Abot A., Bonnafous C., Touchard F. (2012). Effects of cultural conditions on the hemp (*Cannabis sativa*) phloem fibres: Biological development and mechanical properties.

[8] House J. D., Neufeld J., Leson G. (2010). Evaluating the quality of protein from hemp seed (*Cannabis sativa L.*) products through the use of the protein digestibility-corrected amino acid score method.

[9] Bouloc P., Allegret S., Arnaud L. (2012).

[10] Schäfer T., Honermeier B. (2006). Effect of sowing date and plant density on the cell morphology of hemp (*Cannabis sativa* L.).

[11] Amaducci S., Zatta A., Pelatti F., Venturi G. (2008). Influence of agronomic factors on yield and quality of hemp (*Cannabis sativa* L.) fibre and implication for an innovative production system.

[12] Mihoc M., Pop G., Alexa E., Radulov I. (2012). Nutritive quality of Romanian hemp varieties (*Cannabis sativa L.*) with special focus on oil and metal contents of seeds.

[13] Deng C. M., Li J., Sun T., Li C. J. (2007). Occurrence regularity and control measurements of main diseases and insect pests in hemp.

[14] Jiang Y. (2009). China’s water scarcity.

[15] van Bakel H., Stout J. M., Cote A. G. (2011). The draft genome and transcriptome of *Cannabis sativa*.

[16] Gao C., Xin P., Cheng C. (2014). Diversity analysis in *Cannabis sativa* based on large-scale development of expressed sequence tag-derived simple sequence repeat markers.

[17] Hong Y., Zhang W., Wang X. (2010). Phospholipase D and phosphatidic acid signalling in plant response to drought and salinity.

[18] Liu T., Zhu S., Tang Q., Yu Y., Tang S. (2013). Identification of drought stress-responsive transcription factors in ramie (*Boehmeria nivea L. Gaud*).

[19] Yu Y., Zeng L., Yan Z. (2015). Identification of ramie genes in response to *Pratylenchus coffeae* infection challenge by digital gene expression analysis.

[20] Zeng L., Shen A., Chen J. (2016). Transcriptome analysis of ramie (*Boehmeria nivea* L. Gaud.) in response to ramie moth (*Cocytodes coerulea* Guenée) infestation.

[21] Guo Y., Qiu C., Long S. (2017). Digital gene expression profiling of flax (*Linum usitatissimum* L.) stem peel identifies genes enriched in fiber-bearing phloem tissue.

[22] Li H., Li D., Chen A., Tang H., Li J., Huang S. (2016). Characterization of the Kenaf (*Hibiscus cannabinus*) global transcriptome using Illumina Paired-End sequencing and development of EST-SSR markers.

[23] Yang Z., Yan A., Lu R. (2017). De novo transcriptome sequencing of two cultivated jute species under salinity stress.

[24] Huang L., Zhang F., Zhang F. (2014). Comparative transcriptome sequencing of tolerant rice introgression line and its parents in response to drought stress.

[25] Liu Z., Xin M., Qin J. (2015). Temporal transcriptome profiling reveals expression partitioning of homeologous genes contributing to heat and drought acclimation in wheat (*Triticum aestivum* L.).

[26] Xu J., Yuan Y., Xu Y. (2014). Identification of candidate genes for drought tolerance by whole-genome resequencing in maize.

[27] Fan X. D., Wang J. Q., Yang N. (2013). Gene expression profiling of soybean leaves and roots under salt, saline–alkali and drought stress by high-throughput Illumina sequencing.

[28] Bowman M. J., Park W., Bauer P. J. (2013). RNA-Seq transcriptome profiling of upland cotton (*Gossypium hirsutum* L.) root tissue under water-deficit stress.

[29] Xie F., Wang Q., Sun R., Zhang B. (2015). Deep sequencing reveals important roles of microRNAs in response to drought and salinity stress in cotton.

[30] Zhang N., Liu B., Ma C. (2014). Transcriptome characterization and sequencing-based identification of drought-responsive genes in potato.

[31] Yuan G., Yu-Fu W., Cai-Sheng Q., Song-Hua L., Xin D., Dong-Mei H. (2011). Preliminary study on effects of drought stress on physiological characteristics and growth of different hemp cultivars (*Cannabis Sativa* L.).

[32] Hu H., Dai M., Yao J. (2006). Overexpressing a NAM, ATAF, and CUC (NAC) transcription factor enhances drought resistance and salt tolerance in rice.

[33] Love M. I., Huber W., Anders S. (2014). Moderated estimation of fold change and dispersion for RNA-seq data with DESeq2.

[34] Kanehisa M., Sato Y., Kawashima M., Furumichi M., Tanabe M. (2016). KEGG as a reference resource for gene and protein annotation.

[35] Conesa A., Gotz S., Garcia-Gomez J. M., Terol J., Talon M., Robles M. (2005). Blast2GO: a universal tool for annotation, visualization and analysis in functional genomics research.

[36] Shinozaki K., Yamaguchi-Shinozaki K. (2007). Gene networks involved in drought stress response and tolerance.

[37] Reddy A. R., Chaitanya K. V., Vivekanandan M. (2004). Drought-induced responses of photosynthesis and antioxidant metabolism in higher plants.

[38] Cruz de Carvalho M. H. (2014). Drought stress and reactive oxygen species: production, scavenging and signaling.

[39] Zhai H., Wang F., Si Z. (2016). A *myo*-inositol-1-phosphate synthase gene, *IbMIPS1*, enhances salt and drought tolerance and stem nematode resistance in transgenic sweet potato.

[40] Duan J., Zhang M., Zhang H. (2012). *OsMIOX*, a *myo*-inositol oxygenase gene, improves drought tolerance through scavenging of reactive oxygen species in rice (*Oryza sativa* L.).

[41] Yang J., Zhang J., Wang Z., Xu G., Zhu Q. (2004). Activities of key enzymes in sucrose-to-starch conversion in wheat grains subjected to water deficit during grain filling.

[42] Wang Z., Xu Y., Chen T., Zhang H., Yang J., Zhang J. (2015). Abscisic acid and the key enzymes and genes in sucrose-to-starch conversion in rice spikelets in response to soil drying during grain filling.

[43] Thalmann M., Pazmino D., Seung D. (2016). Regulation of leaf starch degradation by abscisic acid is important for osmotic stress tolerance in plants.

[44] Lee K. J. D., Marcus S. E., Knox J. P. (2011). Cell wall biology: perspectives from cell wall imaging.

[45] Cavalier D. M., Lerouxel O., Neumetzler L. (2008). Disrupting two *Arabidopsis thaliana* xylosyltransferase genes results in plants deficient in xyloglucan, a major primary cell wall component.

[46] Nakashima K., Ito Y., Yamaguchi-Shinozaki K. (2009). Transcriptional regulatory networks in response to abiotic stresses in Arabidopsis and grasses.

[47] Fujita Y., Fujita M., Shinozaki K., Yamaguchi-Shinozaki K. (2011). ABA-mediated transcriptional regulation in response to osmotic stress in plants.

[48] Hong Y., Zhang H., Huang L., Li D., Song F. (2016). Overexpression of a stress-responsive NAC transcription factor gene *ONAC022* improves drought and salt tolerance in rice.

[49] Thirumalaikumar V. P., Devkar V., Mehterov N. (2018). NAC transcription factor JUNGBRUNNEN1 enhances drought tolerance in tomato.

[50] Chen D., Chai S., McIntyre C. L., Xue G. P. (2018). Overexpression of a predominantly root-expressed NAC transcription factor in wheat roots enhances root length, biomass and drought tolerance.

[51] Zhu J. K. (2002). Salt and drought stress signal transduction in plants.

[52] Bari R., Jones J. D. G. (2009). Role of plant hormones in plant defence responses.

[53] Umezawa T., Nakashima K., Miyakawa T. (2010). Molecular basis of the core regulatory network in ABA responses: sensing, signaling and transport.

[54] Park S. Y., Fung P., Nishimura N. (2009). Abscisic acid inhibits type 2C protein phosphatases via the PYR/PYL family of START proteins.

[55] Kulik A., Wawer I., Krzywińska E., Bucholc M., Dobrowolska G. (2011). SnRK2 protein kinases—key regulators of plant response to abiotic stresses.

[56] Kim H., Lee K., Hwang H. (2014). Overexpression of *PYL5* in rice enhances drought tolerance, inhibits growth, and modulates gene expression.

[57] Liu L., Hu X., Song J., Zong X., Li D., Li D. (2009). Over-expression of a *Zea mays* L. protein phosphatase 2C gene (*ZmPP2C*) in *Arabidopsis thaliana* decreases tolerance to salt and drought.

[58] Phan T. T., Sun B., Niu J. Q. (2016). Overexpression of sugarcane gene *SoSnRK2.1* confers drought tolerance in transgenic tobacco.

[59] Santiago J., Rodrigues A., Saez A. (2009). Modulation of drought resistance by the abscisic acid receptor PYL5 through inhibition of clade A PP2Cs.

[60] Krzywińska E., Kulik A., Bucholc M., Fernandez M. A., Rodriguez P. L., Dobrowolska G. (2016). Protein phosphatase type 2C PP2CA together with ABI1 inhibits SnRK2.4 activity and regulates plant responses to salinity.

[61] Sun L., Wang Y. P., Chen P. (2011). Transcriptional regulation of *SlPYL*, *SlPP2C*, and *SlSnRK2* gene families encoding ABA signal core components during tomato fruit development and drought stress.

[62] Chen P., Sun Y. F., Kai W. B. (2016). Interactions of ABA signaling core components (SlPYLs, SlPP2Cs, and SlSnRK2s) in tomato (*Solanum lycopersicon*).

[63] Wang Y., Wu Y., Duan C. (2012). The expression profiling of the *CsPYL, CsPP2C* and *CsSnRK2* gene families during fruit development and drought stress in cucumber.

[64] Blatt M. R. (2000). Cellular signaling and volume control in stomatal movements in plants.

[65] Yang L., Ji W., Gao P. (2012). GsAPK, an ABA-activated and Calcium-independent SnRK2-type kinase from *G. soja*, mediates the regulation of plant tolerance to salinity and ABA stress.

[66] Staswick P. E., Serban B., Rowe M. (2005). Characterization of an Arabidopsis enzyme family that conjugates amino acids to indole-3-acetic acid.

[67] Du H., Wu N., Fu J. (2012). A GH3 family member, OsGH3-2, modulates auxin and abscisic acid levels and differentially affects drought and cold tolerance in rice.

