# Supplementary material for: Genome-Wide Expression Profiles of Hemp (Cannabis sativa L.) in Response to Drought Stress
Source: Int J Genomics. 2018 May 15;2018:3057272. doi: 10.1155/2018/3057272 (PMC5976996; doi:10.1155/2018/3057272)
Supplement: Supplementary 1 — Figure S1: RPKM distribution and RPKM density distribution of different genes in the DS and CK hemp. A: box-plot of RPKM. x-axis means sample names, and y-axis means log10(RPKM). B: RPKM distribution. x-axis represent log10(RPKM), and y-axis represents gene density. E1, E2, E3, and E4 represent DS1, DS2, CK1 and CK2, respectively. Figure S2: correlations between two repeats of the CK and DS samples. E1 and E2 represent DS1 and DS2, and E3 and E4 represent CK1 and CK2, respectively. The logarithmic values of (FPKM) for each gene in the two replicates were assigned as coordinate values of the two axes. [file 3057272.f1.pdf]

FIGURE S1

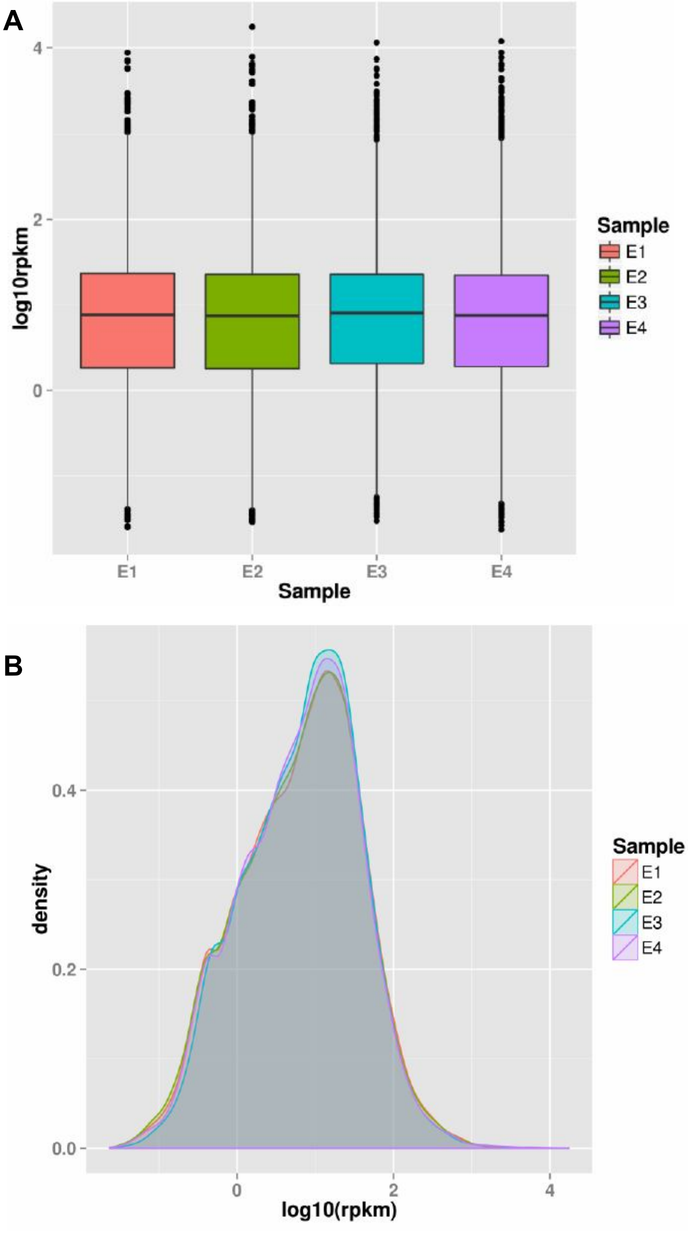

Figure S1: RPKM distribution and RPKM density distribution of different genes in the DS and CK hemp. A: box-plot of RPKM. x-axis means sample names, y-axis means log_{10}(RPKM). **B**: RPKM distribution. x-axis represent log_{10}(RPKM), y-axis represent gene density. E1, E2, E3 and E4 represent DS1, DS2, CK1 and CK2, respectively.

FIGURE S2

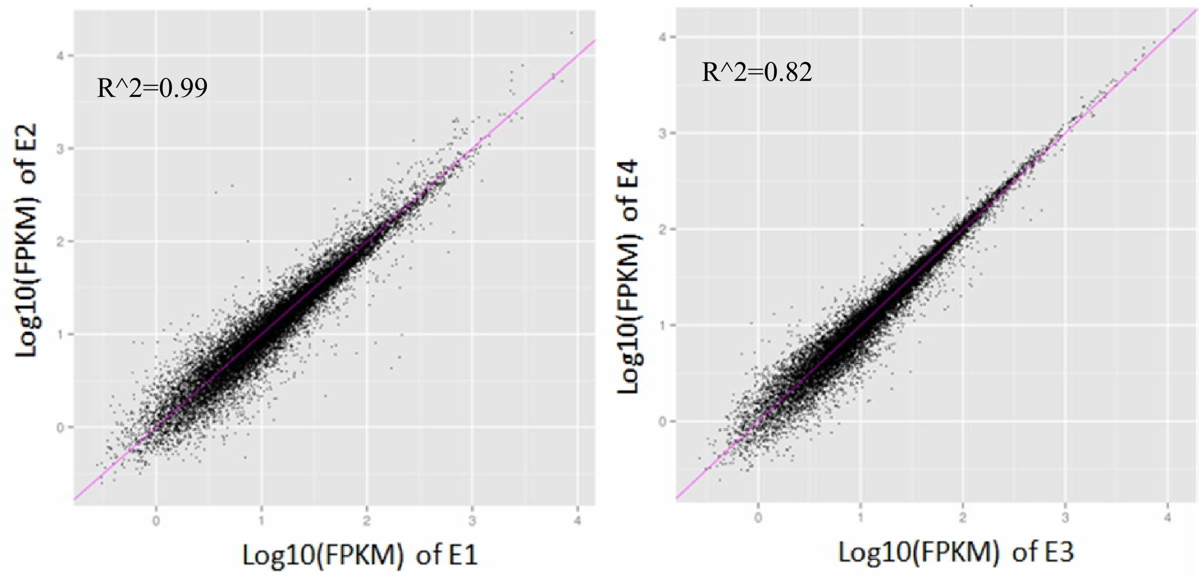

FIGURE S2: Correlations between two repeats of the CK and DS samples. E1 and E2 represent DS1 and DS2, and E3 and E4 represent CK1 and CK2, respectively. The logarithmic values of (FPKM) for each gene in the two replicates were assigned as coordinate values of the two axes.
